# Supplementary material for: Nup98 FG domains from diverse species spontaneously phase-separate into particles with nuclear pore-like permselectivity
Source: eLife. 2015 Jan 6;4:e04251. doi: 10.7554/eLife.04251 (PMC4283134; doi:10.7554/eLife.04251)

```
(*Rational for simulation of influx into an FG particle *)
(* A perfectly spherical FG particle is placed in a far larger buffer reservior . The initial concentration of the mobiles
  species is 1 in the buffer and 0 inside the particle . We simulate the influx of the mobile species with the
  input parameters given below and exploit the spherical symmetry of the system . The system is subdivided
  into concentric sphere shells (n shells for the particle and m shells for the surrounding buffer ). Fluxes
  are computed from concentration differences between neighbouring shells *)

(*Input parameters *)
diameter = 7 (*diameter of the FG particle in µm*);
D1 = 0.1 (*Diffusion constant of mobile species inside the FG particle in µm^2/sec *);
D2 = 50 (*Diffusion constant in free buffer in µm^2/sec*);
PC = 220 (*Partition coeffient FG particle : buffer *);
CaptureEfficiency = 1 (*Probability that a colliding NTR·cargo complex gets captured by the particle *);
time = 1000 (*defines for how many seconds the simulation should run*);
n = 50 (*Subdivision of the FG particle into n concentric and equi-distant shells *);
m = 1000 (*Subdivision of the sourounding buffer compartment into m shells *);
concentration = 1 (*starting concentration of mobile outside the sphere *);

(*Equations for volumes and contact areas*)
(*Thickness of sphere shells *)
h = diameter/(2 * n);

(*Volume V[i] for each sphere shell i is calculated *)
Do[V[i] = (4 * π * h^3 * (i^3 - (i - 1)^3))/3, {i, 1, n + m}];

(*Contact areas A[i] are calcuated for all shell neighbours i and i+1*)
Do[A[i] = 4 * π * h^2 * i^2, {i, 1, n + m}];

(*Equations for fluxes Flux[i,i+1] between neighbouring shells *)
(*Implements Ficks first law of diffusion :
    Flux[i,i+1]=-A*D*(c[i]-c[i+1])/h
  with A being the contact area, D the diffusion constant , and (c[i]-c[i+1])/h the local concentration gradient .*)


(*Shell 1 is set as boundary with zero flux to the outside of the system *)
Flux[0, 1] = 0;

(*Fluxes inside the FG particles with diffusion constant D1*)
Do[Flux[i, i + 1] = (D1 * A[i] * (c[i + 1][t] - c[i][t]))/h, {i, 1, n - 1}];

(*Flux between outermost FG particle shell and adjacent buffer shell. Capture rate for a mobile species
  by the FG particle is given by the diffusion constant inside the buffer (D2) multiplied with Capture
  efficiency . Exit rate from the FG particle equals the entry rate divided by the partition coefficient PC.*)
Flux[n, n + 1] = CaptureEfficiency * (D2 * A[n] (c[n + 1][t] - c[n][t]/PC))/h;

(*Fluxes in free buffer *)
Do[Flux[i, i + 1] = (D2 * A[i] (c[i + 1][t] - c[i][t]))/h, {i, n + 1, n + m - 1}];

(*Shell n+m is set as boundary with zero flux to the outside of the system *)
Flux[n + m, n + m + 1] = 0;
```

(*Assembly of an ordinary differential equation (ODE) system *)

(*ODEs describe how the local concentration of the mobile species changes in each of the shells over time *)

```
Do[ODE[i] = ∂t c[i][t] == (Flux[i, i + 1] - Flux[i - 1, i]) / V[i], {i, 1, n + m}]
ODES = Table[ODE[i], {i, 1, n + m}];
```

(*Setup of initial conditions *)

```
InitialConditions =
  Join[Table[c[i][0] == 0, {i, 1, n}], Table[c[i][0] == concentration, {i, n + 1, n + m}] ];
```

(*Definition of seeked functions *)

```
Functions = Table[c[i], {i, 1, n + m}];
```

(*Numerical solution to the ODE system *)

```
lsg = NDSolve[Join[ODES, InitialConditions], Functions, {t, 0, time}];
```

(*Output of simulation data *)

(*Input of timepoints in seconds *)

```
timepoints = {180, 30, 1};
```

(*Actual output *)

```
nn = Length[timepoints];
Do[timepoint = timepoints[[k]];
  simu[k] = Flatten[Table[{h * i, c[Abs[i] + 1][timepoint] / c[n + m][timepoint]} /. lsg, {i, -n - n, n + n}], 1],
  {k, 1, nn}];
ListLinePlot[Table[Legended[simu[k], StringJoin[ToString[timepoints[[k]]], " sec"]],
  {k, 1, nn}],
 AxesOrigin → {-2 * h * n, -5},
 Frame → True,
 FrameLabel →
  {{"Concentration of mobile species", None}, {"µm from particle center", None}},
 GridLines → Automatic,
 FrameStyle → Thickness[0.0015],
 BaseStyle → {FontFamily → "Helvetica", FontSize → 16},
 ImageSize → {400, 300},
 PlotRange → {{-diameter, +diameter}, {-10, PC + 30}}]
```

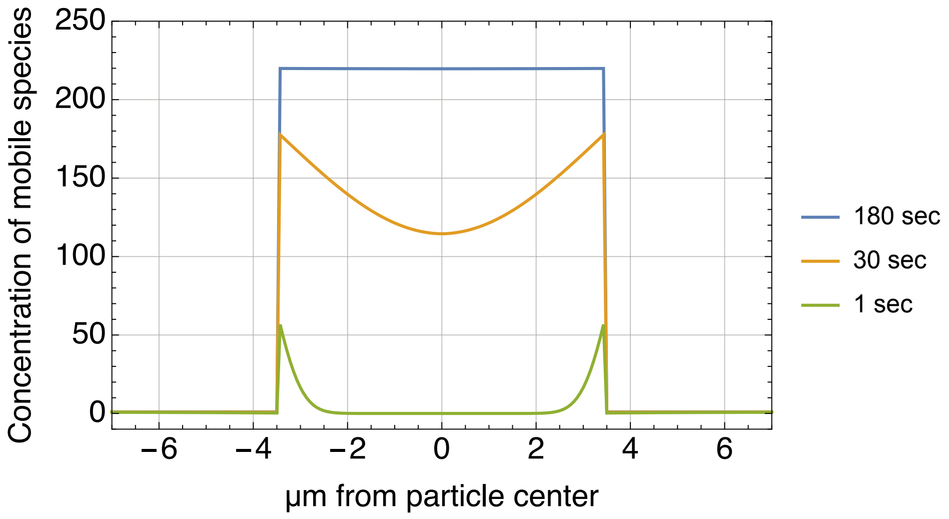

Supplement: Supplementary file 1. — Mathematica program code used for the simulations in Figure 3. DOI: http://dx.doi.org/10.7554/eLife.04251.022 [file elife04251s001.pdf]
