# Supplementary material for: Nup98 FG domains from diverse species spontaneously phase-separate into particles with nuclear pore-like permselectivity
Source: eLife. 2015 Jan 6;4:e04251. doi: 10.7554/eLife.04251 (PMC4283134; doi:10.7554/eLife.04251)

Homo sapiens Nup98

500 residues (443 without GLEBS domain)

MFNKSFGTPFGGGTGGFGTTSTFGQNTGFGTTSGGAFGTSAFGSSNNTGGLFGNSQTKPGGLFGTSSFSQP
ATSTSTGFGFGTSTGTANTLFGTASTGTSLFSSQNNAFAQNKPTGFGNFGTSTSSGGLFGTTNTTSNPFGS
TSGSLFGPSSFTAAptgttikfnpptgtdtmvkagvstnistkhqcitamkeyesksleelrledyqanrk
GPQNQVGAGTTTGLFGSSPATSSATGLFSSSTTNSGFAYGQNKTAFGTSTTGFGTNPGGLFGQQNQQTTSL
FSKPFGQATTTQNTGFSFGNTSTIGQPSTNTMGSFGVTQASQPGGLFGTATNTSTGTAFGTGTGLFGQTNT
GFGAVGSTLFGNNKLTTFGSGTTSAPSFGTTSGGLFGFGTNTSGNSIFGSKPAPGTLGTGLGAGFGTALGA
GQASLFGNNQPKIGGPLGTGAFGAPGFNTTTATLGFGAPQAPVALTDPNASAAQQAVLQQHINSLTYSPFG
DS

Localization of GLEBS domain:

Distribution of all 39 FG dipeptides:

Distribution of remaining hydrophobic residues:

Distribution of NQ residues:

Distribution of charged residues:

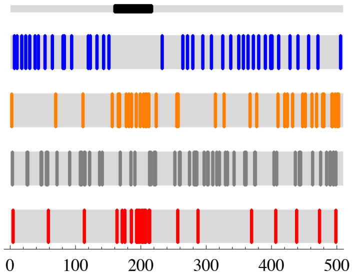

Branchiostoma floridae Nup98

479 residues (427 without GLEBS domain)

MFGQQKTPFGGTTGFGTGAFGTSSFGATQTPATGGLFGGTATNTGTGLFGGTSFGTPSTSTSTFGGFGTST
TQTGGGLFGTSTSTAGTGLFATPQQQTAPFGAANKTGFGGFGTQTSTAATGTGLFGATQQTPSLFGGGQTT
STGLFGAVGGIAAGTNGTTvkfnpvsgsdtmmkngvsqnirtahqcitamkeyetksleelrvedylanrk
GGSTGTTAMFGATATPQTGGGLFGNTATTTSTTGFTFGKAAFGTGQTQAKGATGFGTTSTGTGLFGQTQTT
QAGGLFASPFGGTATTTTPSTGFSFGQTNTGTGLFGQTQQKTGLFGQPTTQTTGLFGTPSTTTTTGFGTTF
GTGTFGTQNQAGGLFGANKAPTFGATTTTSTTGGLFGNTATNTGGLFGQNKPGLTLGLGTGFGTGAFGTTT
TSTGTSLFGPKPTNTFGAGLGTGLGAGLGTLGTGSIFGNTGLGAGIGTGTTG

Localization of GLEBS domain:

Distribution of all 40 FG dipeptides:

Distribution of remaining hydrophobic residues:

Distribution of NQ residues:

Distribution of charged residues:

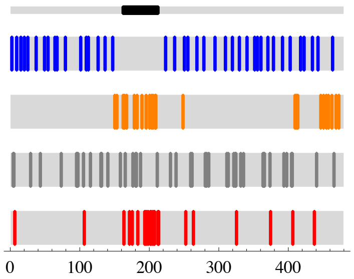

Drosophila melongaster Nup98

581 residues (528 without GLEBS domain)

MFGGAKPSFGATPAATSFGGFSGTTTTTPFGQSAFGKPAAPAFGNTSTFAAQPAQQSLFGAAATPAQPAGG
LFGANTSTGFGSTATAQPTAFGAFSQPQQTSNIFGSTQTAASTSLFGQSTLPAFGAAKPTMTAFGQTAAAQ
PTGSLFGQPAAATSTTGFGGFGTSAPTTTNVFGSGTASAFAQPQATAVGASGVNTGTAvakyqptigtdtl
mksgqansvntkqhcitamkefegksleelrledymcgrkGPQAGNAPGAFGFGAQVTQPAQPASGGLFGS
TAQPSTGLFGQTVTENKSMFGTTAFGQQPATNNAFGAATQQNNFLQKPFGATTTTPFAAPAADASNPFGAK
PAFGQGGSLFGQAPATSAAPAFGQTNTGFGGFGTTAGATQQSTLFGATPAADPNKSAFGLGTAASAATTGF
GFGAPATSTAGGGLFGNKPATSFAAPTFGATSTASTPFSNFGLNTSTAATGGGLFNSGLNKPATSGFGGFG
ATSAAPLNFNAGNTGGSLFGNTAKPGGGLFGGGTTTLGGTGAAPTGGLFGGGTTSFGGVGGSLGGGGFGMG
TNNSLTGGIMGA

Localization of GLEBS domain:

Distribution of all 46 FG dipeptides:

Distribution of remaining hydrophobic residues:

Distribution of NQ residues:

Distribution of charged residues:

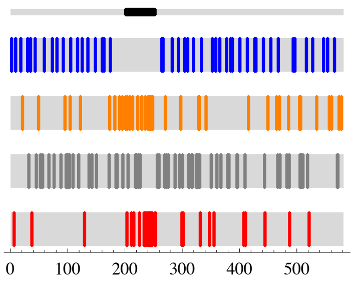

Caenorhabditis elegans Nup98

494 residues (442 without GLEBS domain)

MFGQNKSFGSSSFGGGSSGSGLFGQNNQNNQNKGLFGQPANNSGTTGLFGAAQNKPAGSIFGAASNTSSIF
GSPQQPQNNQSSLFGGGQNNANRSIFGSTSSAAPASSSLFGNNANNTGTSSIFGSNNNAPSGGGLFGASTV
SGTTvkfeppissdtmmrngttqtistkhmcisamskydgksieelrvedyianrkAPGTGTTSTGGGLFG
ASNTTNQAGSSGLFGSSNAQQKTSLFGGASTSSPFGGNTSTANTGSSLFGNNNANTSAASGSLFGAKPAGS
SLFGSTATTGASTFGQTTGSSLFGNQQPQTNTGGSLFGNTQNQNQSGSLFGNTGTTGTGLFGQAQQQPQQQ
SSGFSFGGAPAATNAFGQPAAANTGGSLFGNTSTANTGSSLFGAKPATSTGFTFGATQPTTTNAFGSTNTG
GGLFGNNAAKPGGLFGNTTNTGTGGGLFGSQPQASSGGLFGSNTQATQPLNTGFGNLAQPQIVMQQQ

Localization of GLEBS domain:

Distribution of all 36 FG dipeptides:

Distribution of remaining hydrophobic residues:

Distribution of NQ residues:

Distribution of charged residues:

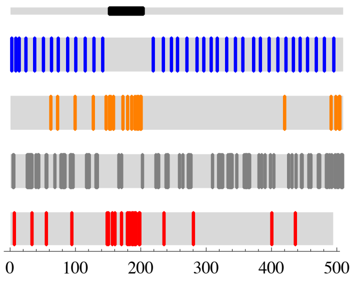

Saccharomyces cerevisiae Nup100

578 residues (578 without GLEBS domain)

MFGNNRPMFGGSNLSFGSNTSSFGGQQSQQPNSLFGNSNNNNNSTSNNAQSGFGGFTSAAGSNSNSLFGNN
NTQNNGAFGQSMGATQNSPFGSLNSSNASNGNTFGGSSSMGSFGGNTNNAFNNNSNSTNSPFGFNKPNTGG
TLFGSQNNNSAGTSSLFGGQSTSTTGTFGNTGSSFGTGLNGNGSNIFGAGNNSQSNTTGSLFGNQQSSAFG
TNNQQGSLFGQQSQNTNNAFGNQNQLGGSSFGSKPVGSGSLFGQSNNTLGNTTNNRNGLFGQMNSSNQGSS
NSGLFGQNSMNSSTQGVFGQNNNQMQINGNNNNSLFGKANTFSNSASGGLFGQNNQQQGSGLFGQNSQTSG
SSGLFGQNNQKQPNTFTQSNTGIGLFGQNNNQQQQSTGLFGAKPAGTTGSLFGGNSSTQPNSLFGTTNVPT
SNTQSQQGNSLFGATKLTNMPFGGNPTANQSGSGNSLFGTKPASTTGSLFGNNTASTTVPSTNGLFGNNAN
NSTSTTNTGLFGAKPDSQSKPALGGGLFGNSNSNSSTIGQNKPVFGGTTQNTGLFGATGTNSSAVGSTGKQ
NNNTLNVGT

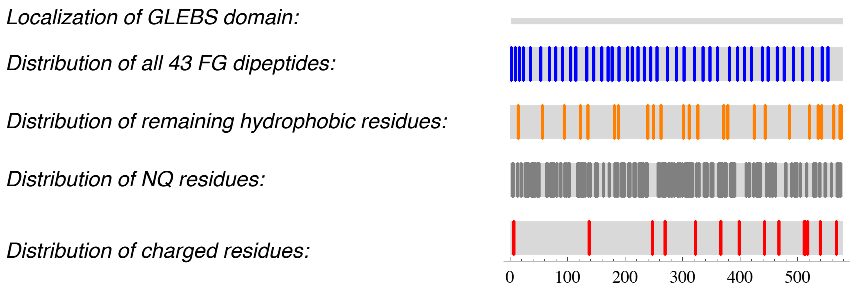

Saccharomyces cerevisiae Nup116

737 residues (680 without GLEBS domain)

MFGVSRGAFPSATTQPFGSTGSTFGGQQQQQQPVANTSAFGLSQQTNTTQAPAFGNFGNQTSNSPFGMSGS
TTANGTPFGQSQLTNNNASGSIFGGMGNNTALSAGSASvvpnstagtsikpfttfeekdpttgvinvfqsi
tcmpeyrnfsfeelrfqdyqagrkFGTSQNGTGTTFNNPQGTTNTGFGIMGNNNSTTSATTGGLFGQKPAT
GMFGTGTGSGGGFGSGATNSTGLFGSSTNLSGNSAFGANKPATSGGLFGNTTNNPTNGTNNTGLFGQQNSN
TNGGLFGQQQNSFGANNVSNGGAFGQVNRGAFPQQQTQQGSGGIFGQSNANANGGAFGQQQGTGALFGAKP
ASGGLFGQSAGSKAFGMNTNPTGTTGGLFGQTNQQQSGGGLFGQQQNSNAGGLFGQNNQSQNQSGLFGQQN
SSNAFGQPQQQGGLFGSKPAGGLFGQQQGASTFASGNAQNNSIFGQNNQQQQSTGGLFGQQNNQSQSQPGG
LFGQTNQNNNQPFGQNGLQQPQQNNSLFGAKPTGFGNTSLFSNSTTNQSNGISGNNLQQQSGGLFQNKQQP
ASGGLFGSKPSNTVGGGLFGNNQVANQNNPASTSGGLFGSKPATGSLFGGTNSTAPNASSGGIFGSNNASN
TAATTNSTGLFGNKPVGAGASTSAGGLFGNNNNSSLNNSNGSTGLFGSNNTSQSTNAGGLFQNNTSTNTSG
GGLFSQPSQSMAQSQNALQQQQQQQR

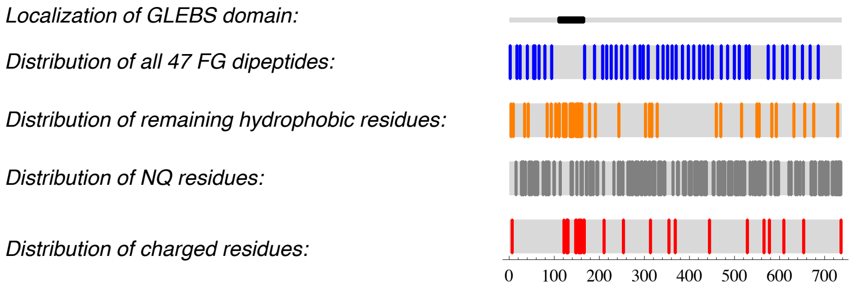

Dictyostelium discoideum Nup220

719 residues (669 without GLEBS domain)

MFGGQFGSFGAKPAATASPFGAPSAAPTTSLFGSTAPSSGFGGFGSTAQTTQPTTGGFGGFGGFGGATTTQ
QPAASPFGGGGTGGSGLFGSSAQTTTQQPGASPFGGGFGTTTTTTTQQPGASPFGGTGGGLFGSSAQTTTQ
QQGASPFGGFGGATTTQPSLLSGATGGFGGFGGSTTSGTQLGGGGGATSGAFGGSSSPFGGSGGATTSSPF
GGGGGSFGATTQkqygtpipyqqttiegntfvsisampqyndrsfeelrfedithrkdivykTGGGSGGGN
SLFGSTPTTQPSSPFGAQTTTQTTGGLFGGQTTTSPFGGQTSATPGSSLFGSTQPTQQQTSGGLFGSVQPT
QQQAGGGLFGSMPSTGGSSLFGSTQPTQQQTGGAQPTQSLFGGQTQTTTSPFGSQTSTPFGQPQQTNTGSG
LFGAQQTQQTNTGGGLFGAQPTQQTSGGGLFGTQPTSGTGLFGTSPTAGGTGLFGTTQPTSQGTGLFGTTQ
PTTQGTGLFGTSPTSGTGLFGSTPTSGTGLFGSTPTSGTGLFGSAQPPQNQQSQTSLFGNTGTGATNTGTG
LFGSAQPSSNPGGGLFGSAQPSTTTGGLFGSNQPTAQPTTSLFGNTTGSVGGLGATPNITSGLFGSNPAQT
GGLFGSTQPTTQTSLFGNTGSTGGLGAQNGGGLFGNLSQPTATAGQGLSGGLFGNLSQPTATAGQGLSSGG
LFGNTLLG

Localization of GLEBS domain:

Distribution of all 56 FG dipeptides:

Distribution of remaining hydrophobic residues:

Distribution of NQ residues:

Distribution of charged residues:

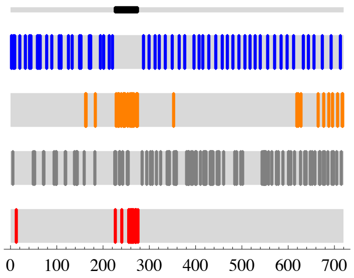

Arabidopsis thaliana Nup98B

668 residues (620 without GLEBS domain)

MFGSSNNNPFGQSSISSPFGTQTHSLFGQTNNNASNNPFATKPFGTSTPFGAQTGSSMFGGTSTGVFGAPQ
TSSPFGASPQAFGSSTQAFGASSTPSFGSSNSPFGGTSTFGQKSFGLSTPQSSPFGSTTQQSQPAFGNSTF
GSSTPFGASTTPAFGASSTPAFGVSNTSGFGATNTPGFGATNTTGFGGSSTPGFGASSTPAFGSTNTPAFG
ASSTPLFGSSSSPAFGASPAPAFGSSGNAFGNNTFSSGGAFGSSSTPTFGASNTSAFGASSSPSFNFGSSP
AFGQSTSAFGSSSFGSTQSSLGSTPSPFGAQGAQASTSTFGGQSTIGGQQGGSrvipyapttdtasgtesk
serlqsisampahkgknmeelrwedyqrgdkGGQRSTGQSPEGAGFGVTNSQPSIFSTSPAFSQTPVNPTN
PFSQTTPTSNTNFSPSFSQPTTPSFGQPTTPSFRSTVSNTTSVFGSSSSLTTNTSQPLGSSIFGSTPAHGS
TPGFSIGGFNNSQSSPLFGSNPSFAQNTTPAFSQTSPLFGQNTTPALGQSSSVFGQNTNPALVQSNTFSTP
STGFGNTFSSSSSLTTSISPFGQITPAVTPFQSAQPTQPLGAFGFNNFGQTQIANTTDIAGAMGTFSQGNF
KQQPALGNSAVMQPTPVTNPFGTLPALPQ

Localization of GLEBS domain:

Distribution of all 52 FG dipeptides:

Distribution of remaining hydrophobic residues:

Distribution of NQ residues:

Distribution of charged residues:

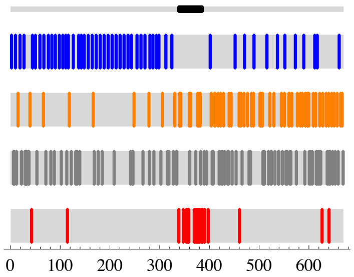

Tetrahymena thermophila macronuclear Nup98A

666 residues (633 without GLEBS domain)

MFGNTGGGGLFGNTQTQQTGGGLFGQPQQTQFGQTGATGGGLFGGATNTFGGGGGGGLFGGNNNQQTNPTA
GGGIFGQGTTGLGGAPAQTGGGLFGAPQNNQGGGLFGGGTTTGGGMFGNQANTQTGGGGLFGGPSQPTTQP
PAFSLNNPTTGGGGLFGQPANTMGGNNGGLFGGQTNSFGANNNMLGNNNRPQGAGIFGGATTTAPTGNTGM
FGGIGANNGGGGLFGMNNTNTNPTGGFGATNPTAGGGGLFGGGATTTGGGGLFGGGNTQGGGLLGTANTTA
GGLLGGGFNMNNNTGGILGQTNNQFGLGSFGTNNNAAAAPFQPKASANGvltkpneknlcyaisngtdfci
felaltqrklvkAGQLKPGAQQAGGMFGQPAQGGNGLFGGGGAATTTPFGGAQNGNLFGGQNTQAQGGGLF
GAPVNNAATGAGGGLFGAKPAATTTGGGLFGQMPAQTGGFLGNTATQPAGGGLFGGATTTQAPGGGGGGGL
FGGNTTAATTGGGLFGGNTQTGGATGGLFGGQQPNNQGGLFLNTGNANNANTGGGLFGGATTTPATGGGLF
GGSTNTQPGLATGGGLFGNNQGASQPAAQGGLFGGAAPQQNSLFGGATAGGQTGGLFGGATGATQQQGGGL
FGQTASNPTQGGGLFGAANPGLGGAAA

Localization of GLEBS domain:

Distribution of all 42 FG dipeptides:

Distribution of remaining hydrophobic residues:

Distribution of NQ residues:

Distribution of charged residues:

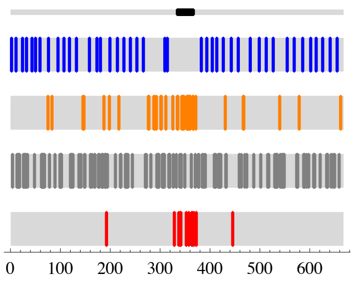

Trypanosoma brucei Nup158

565 residues (565 without GLEBS domain)

MSAGFGGGFGQPAATGFGQQPTGGFGQAPQGGAFGQVAPAATGFGQPSQSAVTGGFGQTNTGGFGQPAATG
FGQPAQGAVTGGFGQTNTGGFGQPAATGFGQPAQSAVTGGFGQTNTGGFGQPAQGGFGQTAAAANAFGQAG
PSGGFGQTNTGGFGQQSNSGFGQAGRGATAGFGQPGTGFGQPATGGFGQATSASPFGQAAAGRGVGGGFGT
AAGTVGGFGQPAATGGFGQTATTGGFGQPAQGAAAGGFGQPATGGFGQATSASPFGQAAAGRGVGGGFGTA
AGTVGGFGQPAAPGGFGQTATAGGFGQPARGAAAGGFGQPATGGFGQATSASPFGQAAAGRGVGGGFGTAA
GTVGGFGQPAATGGFGQTATTGGFGQPAQGANTFGQGTPSAGGFGQAGRGVTGGFGQTGVTGGFGQTATTG
GFGQPAQGAATGGFGQAGRGAADGFGRPAQGAAAGGFGQPATGGFGQATSASPFGQAAAGRGVGGGFGTAA
GTVGGFGQPAAPGGFGQTATTGGFGQPGRGAAAGGFGQPATGGLGLAGGSGFGAAAGAGGFGQQSTAS

Localization of GLEBS domain:

Distribution of all 58 FG dipeptides:

Distribution of remaining hydrophobic residues:

Distribution of NQ residues:

Distribution of charged residues:

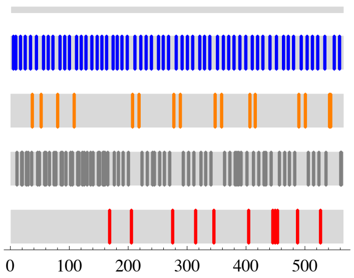

Supplement: Supplementary file 2. — Amino acid sequences of the studied Nup98 FG domains. DOI: http://dx.doi.org/10.7554/eLife.04251.023 [file elife04251s002.pdf]
